# *ROQUIN/RC3H1* Alterations Are Not Found in Angioimmunoblastic T-Cell Lymphoma

**DOI:** 10.1371/journal.pone.0064536

**Published:** 2013-06-25

**Authors:** Tiphanie Auguste, Marion Travert, Karin Tarte, Patricia Amé-Thomas, Catherine Artchounin, Nadine Martin-Garcia, Aurélien de Reynies, Laurence de Leval, Philippe Gaulard, Marie-Hélène Delfau-Larue

**Affiliations:** 1 INSERM U955, Immunologie et Oncogenèse des Tumeurs Lymphoïdes, Créteil, France; 2 Faculté de Médecine, Université Paris-Est, Créteil, France; 3 INSERM U917, Microenvironnement et Cancer, Rennes, France; 4 Programme Cartes d'Identité des Tumeurs, Ligue Nationale Contre le Cancer, Paris, France; 5 Service de Pathologie Clinique, Institut Universitaire de Pathologie, Lausanne, Switzerland; 6 Département de Pathologie, Groupe Hospitalier Henri Mondor–Albert Chenevier, Créteil, France; 7 Laboratoire d'Immunologie Biologique, Assistance Publique–Hôpitaux de Paris (AP-HP), Groupe Henri-Mondor Albert-Chenevier, Créteil, France; Mayo Clinic, United States of America

## Abstract

Angioimmunoblastic T-cell Lymphoma (AITL) is one of the most frequent T-cell lymphoma entities. Follicular helper T lymphocytes (TFH) are recognized as the normal cellular counterpart of the neoplastic component. Despite a clonal T-cell feature and few described recurrent cytogenetic abnormalities, a driving oncogenic event has not been identified so far. It has been recently reported that in mice, heterozygous inactivation of Roquin/Rc3h1, a RING type E3 ubiquitine ligase, recapitulates many of the clinical, histological, and cellular features associated with human AITL. In this study we explored whether ROQUIN alterations could be an initial event in the human AITL oncogenic process. Using microarray and RT-PCR analyses, we investigated the levels of ROQUIN transcripts in TFH tumor cells purified from AITL (n = 8) and reactive tonsils (n = 12) and found similar levels of ROQUIN expression in both. Moreover, we also demonstrated that ROQUIN protein was expressed by AITL TFH (PD1+) cells. We then analysed ROQUIN coding sequence in 12 tumor cell-rich AITL samples and found no mutation in any of the samples. Finally, we analysed the expression of MiR101, a putative partner of ROQUIN involved in the modulation of ICOS expression and found similar levels of expression in tumor and reactive TFH. Altogether, this study shows that neither alteration of ROQUIN gene nor deregulation of miR101 expression is likely to be a frequent recurrent event in AITL.

## Introduction

Angioimmunoblastic T-cell Lymphoma (AITL) is a distinct T-cell lymphoma entity [1] originally described as a dysimmune condition [2]. It usually manifests with generalized lymphadenopathy, hepatomegaly, splenomegaly, fever, sweats, and skin rash and is frequently associated with clinical and biological auto-immune manifestations [3]. A clonal T-Cell Receptor gene rearrangement is detected in around 80% of the cases [4], [5], and few recurrent cytogenetic abnormalities have been reported (reviewed in [6]). Recently, we have reported mutations in isocitrate dehydrogenases 2 (IDH2) [7] and Ten-Eleven Translocation 2 (TET2) [8] genes in AITL, two genes involved in epigenetic gene regulation, but to date, no driving oncogenic event has been identified. We and others have shown that Follicular Helper T (T_FH_) cells are the normal cellular counterpart of the neoplastic component of AITL [9]–[12]. T_FH_ cells constitute a specialized subset of T cells which allows the selection of high-affinity B lymphocytes within germinal centers and provide helper function for antibody production [13]. Human T_FH_ cells express high levels of BCL6, PD1, ICOS, the chemokine CXCL13 and its receptor (CXCR5) and secrete the cytokine IL-21 [14]–[18].

Recently, a mouse model has been proposed for AITL [19]. It recapitulates many of the clinical and pathological features associated with AITL, including lymphadenopathy, hypergammaglobulinemia and accumulation/expansion of clonal T_FH_ cells. This phenotype is specifically linked to heterozygous *Roquin/Rc3h1* point mutation (sanroque allele) in T cells [20]. Roquin, a RING-type E3 ubiquitin ligase family member, has been previously identified as a regulator of autoimmune responses in mice [20].

We thus hypothesized that in human, *ROQUIN/RC3H1* alterations could occur as an initial event of the AITL oncogenic process, leading to T_FH_ accumulation or proliferation prone to subsequent transforming events.

## Material and Methods

The present study was approved by the institutional review board “Comité de Protection des Personnes, Créteil, France» (CPP 09–008). Written consent was obtained from patients with lymphoma. Reactive human tonsils were collected from children undergoing routine tonsillectomy. Oral information was given to parents. A consent form attesting the oral consent was signed by the surgeon and given to the research team with tonsils.

### Cell samples and AITL tissues

**Figure 1:**
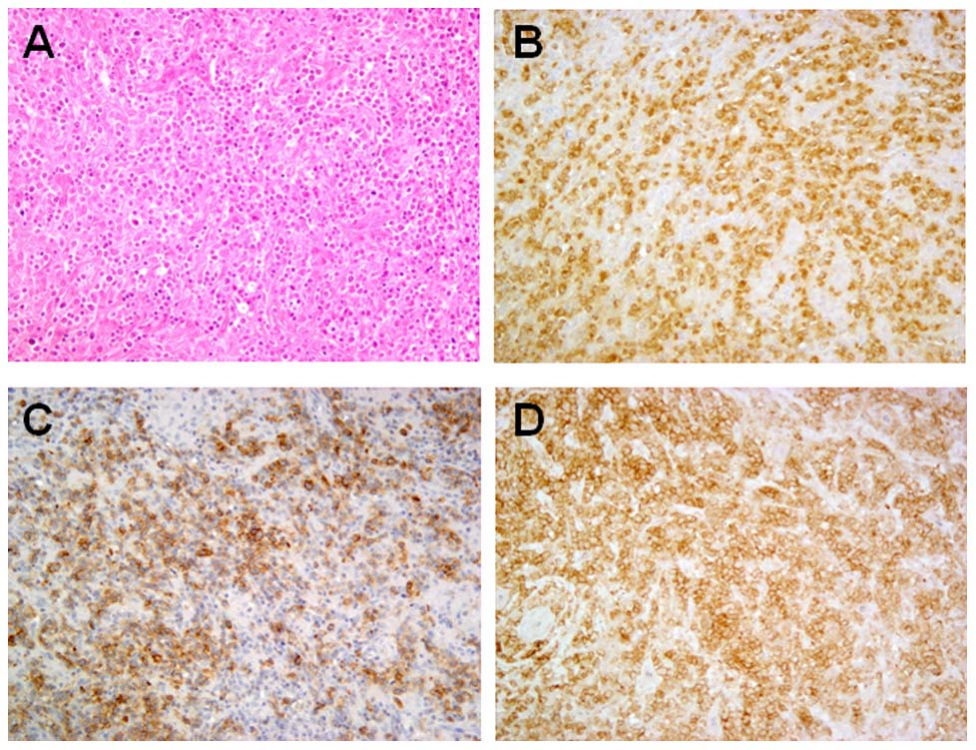
Illustrative case of AITL rich in tumor cells. Diffuse proliferation of large neoplastic cells surrounded by inflammatory cells (plasma cells, eosinophils) and vascular hyperplasia (hematoxylin-eosin, original magnification ×20) (A). Numerous neoplastic cells highlighted by positivity for T_FH_ markers CXCL13 (B), ICOS (C) and PD1(D) (immunoperoxidase, original magnification ×20).

Normal cell subsets were isolated from reactive human tonsils. Briefly, mononuclear cells were isolated by mechanical disruption followed by Ficoll-hypaque density gradient centrifugation. T_FH_ cells were purified after depletion of CD19, CD8, CD14 and CD16-positive cells with magnetic beads (Milteny Biotec, Paris, France), by cell sorting of CD4-FITC, CXCR5-PE and ICOS-PC7 triple-positive cells on Mo-Flo legacy (Beckman Coulter, Villepinte, France). Tonsil CD4^+^, CD8^+^ T-cells and B-cells were purified by positive selection with antibodies directed against CD4, CD8, and CD19 respectively (Milteny Biotec, Paris, France). Neoplastic T_FH_ cells were isolated from cryopreserved mononuclear cell suspensions of AITL lymph node biopsies, through a one-step CD4-FITC, CXCR5-PE and ICOS-PC7 cell sorting. Twelve AITL tumor frozen tissue samples were selected on the basis of high tumor cell content. After complete immunostaining for T_FH_ markers including PD1, ICOS and CXCL13, a semi-quantitative evaluation of tumor cells was performed as previously described [21] and cases with more than 50% tumor cells were selected for *ROQUIN* sequence analyses (Figure 1).

### Immunohistochemistry

For *in situ* evaluation of *ROQUIN* expression, deparaffinised tissue sections of 8 AITL samples were stained with a polyclonal antibody (Novius biologicals NBP1-89590, Cambridge, United Kingdom) using a Vectastain immunoperoxidase method (Vector Labs, Peterborough, UK) and revealed with Diaminobenzidine (DAB). Specificity of the antibody was validated using NIH3T3 transfected with human full length *ROQUIN* cDNA (data not shown). The distribution and phenotypic characteristics of ROQUIN-positive cells *in vivo* was explored by double immunostainings for ROQUIN and either PAX5 (as a B-cell marker) and PD1 (as T_FH_ marker). Briefly, cases were first stained for ROQUIN using an immunoperoxidase method (Vectastain), then for PAX5 ((DakoCytomation, Glostrup, Denmark) or PD1 (ABCAM, Cambridge, UK) using a Vectastain phosphatase alkaline method (Vector Labs) and revealed with Naphtol fast red. Images were captured with a Zeiss Axioskop2 microscope (Zeiss). Photographs were taken with an Olympus DP70 camera. Images were acquired with Olympus DP Controller and images were processed with Adobe Photoshop Version 7.0 (Adobe Systems).

### Microarray and RT-PCR analysis

Total RNAs extracted using TRIZOL reagent (Invitrogen, Carlsbad, CA) were used either for microarray procedures on HG-U133 plus 2.0 Affymetrix GeneChip array) as previously described [12] or for microRNA gene expression profiling on Agilent Human v3 miRNA microarray (G4471A, Agilent, Santa Clara CA). Analyses of gene expression profiles focused on probesets matching to *ROQUIN* (228996_at), *ICOS* (210439_at), and on miR101 (has-miR-101). The levels of *ROQUIN* and *ICOS* transcripts in reactive and neoplastic T_FH_ were determined by TaqMan quantitative reverse-transcriptase PCR (qRT-PCR; Applied Biosystems) on Light Cycler 480, after normalization to *HPRT* mRNA, and compared to purified reactive T_FH_, according to the 2^ΔΔCT^ method.

### Sequence analyses

*ROQUIN* cDNA was amplified by PCR in 3 fragments encompassing the coding sequence. Direct sequencing of PCR products was performed for the first two 5′ fragments. A cloning phase was necessary for exons 16 to 19 sequencing due to alternative splicing. Sequences were obtained on a 3130X1 genetic analyzer (Applied biosystems) and compared with the *ROQUIN* reference sequence (GenBank accession number NM_172071) using seqscape software. PCR and sequencing primers are available upon request. It has been established that this sequencing method allows the detection of a mutated allele when it represents 10% or more of the total amplified alleles [22].

## Results and Discussion

### The levels of *ROQUIN* transcripts are similar in neoplastic and reactive T_FH_ cells

**Figure 2:**
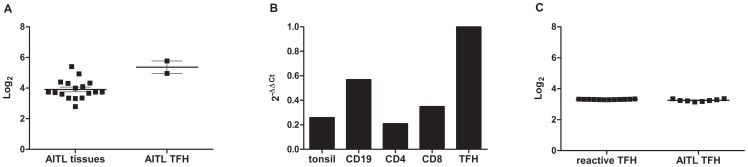
*ROQUIN* expression in human reactive and tumoral lymphoid samples. Levels of *ROQUIN* transcripts determined by gene expression profiling of 17 AITL tumor tissue samples and 2 AITL cell suspensions enriched in tumor cells (≥50%) samples as previously reported [10]. (probe-set 228996_at): *ROQUIN* transcripts level is slightly higher in enriched tumor cell sample (P = 0,0067 unpaired t-test) (A). *ROQUIN* mRNA levels determined by quantitative RT-PCR in reactive tonsils; total extract (n = 1), CD4^+^ (n = 2), CD8^+^ (n = 2), or CD19^+^ (n = 2) lymphocytes. Results were normalized by HPRT and compared to reactive purified TFH cells as calibrator: reactive CD4- CD8- and CD19-positive subsets display heterogeneous levels of *ROQUIN* mRNA (B). *ROQUIN* mRNA levels [(228996_at) probeset] in purified reactive (n = 12) and neoplastic T_FH_ cells (n = 8). T_FH_ cells were purified from 12 reactive tonsils and 8 AITL lymph nodes, RNA was extracted and whole genome expression was analysed on HG-U133 plus 2.0 Affymetrix GeneChip arrays. Similar levels of *ROQUIN* transcript are observed (C).

The analyses of *ROQUIN* probesets in our previously published transcriptomic dataset [12] disclosed the presence of *ROQUIN* transcripts in 17/17 AITL tissue samples with a slightly higher level in the two AITL cell-sorted samples enriched in tumor cells (n = 2) (Figure 2A). However, as reactive T (CD4, CD8) and B (CD19) cell subsets also contain *ROQUIN* mRNA (Figure 2B) and may thus compound the precise analysis of *ROQUIN* level of expression in tumor T_FH_, we thus performed additional transcriptomic analyses of CD4^+^/CXCR5^+^/ICOS^+^ sorted T_FH_ cells obtained from reactive tonsils (n = 12) and AITL (n = 8) on Affymetrix microarray. Similar levels of *ROQUIN* transcripts were observed in T_FH_ purified either from reactive tonsils or from AITL lymph nodes (Figure 2C), thus excluding the hypothesis of a ROQUIN extinction by promoter alteration or gene expression dysregulation in AITL.

### *ROQUIN* protein is expressed by AITL tumor cells

**Figure 3:**
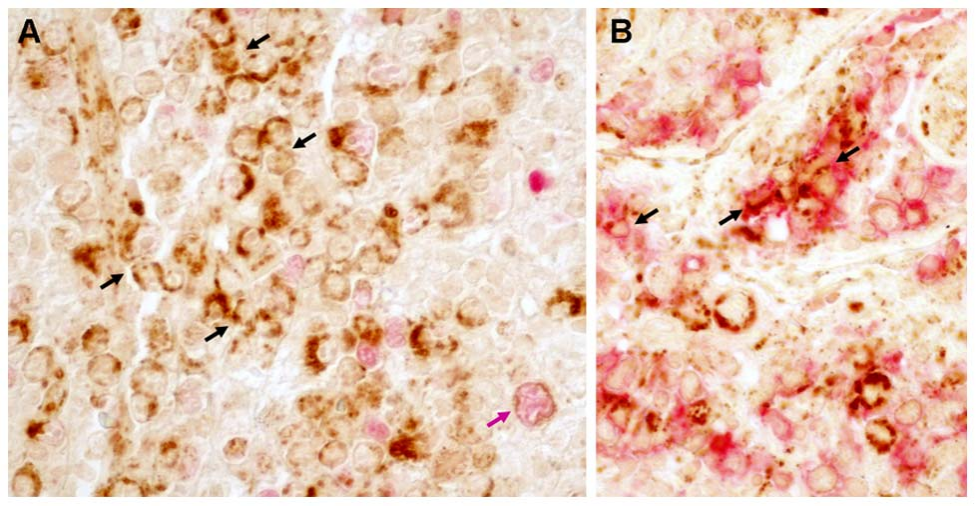
Immunohistochemical detection of ROQUIN in AITL. Among the many cells showing a cytoplasmic granular staining for ROQUIN (brown), a few are PAX5-positive large cells (B-immunoblasts) (pink arrow) whereas most of them are small to medium-sized PAX5-negative lymphoid cells forming small aggregates, corresponding to neoplastic cells of AITL (black arrows) (A). In addition, these aggregates of medium-sized ROQUIN- positive cells (brown, granular staining) co expressed the T_FH_-associated marker PD1 (red, membrane staining) (B). Double immunohistochemistry, original magnification ×250.

*In situ* evaluation of the pattern of ROQUIN expression was performed by immunohistochemistry. In all eight AITL cases investigated, numerous cells showing a granular cytoplasmic staining were observed. These comprised scattered large cells resembling B-blasts, smaller lymphocytes and many small to medium-sized atypical cells suggestive of the neoplastic cell component, as well as endothelial cells (Figure 3A). Double immunostainings performed in 4 cases demonstrated that most ROQUIN-positive cells were PAX5-negative and that many of them expressed PD1, therefore sharing the characteristic morphological and phenotypic features of neoplastic T_FH_ cells (Figure 3B). Furthermore, the observed granular cytoplasmic staining is compatible with Roquin localization in P bodies or stress granules as reported in the mouse [23], [24].

### *ROQUIN* coding sequence is not mutated in human AITL

We next investigated the presence of missense mutations in *ROQUIN* coding sequence. The 3402 bp *ROQUIN* coding sequence was obtained from 12 AITL samples with a high tumor load as well as normal CD4^+^ T cells sorted from 2 reactive tonsils. In contrast to Sanroque mice that develop a TFH cell lymphoproliferative disorder with several symptoms of AITL including auto-immune manifestations and organomegaly as a result of Roquin mutations [19], no mutation was found in any of the AITL patients.

### ICOS and miR101 expression are similarly expressed in reactive and AITL T_FH_

**Figure 4:**
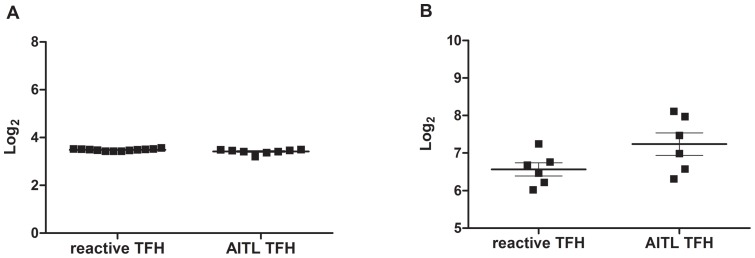
Expression of ICOS and miR101 expression in human reactive and neoplastic T_FH_ cells. Analyses of ICOS expression (210439_at). The level of ICOS mRNA expression is maintained even in the presence of *ROQUIN* transcripts both in human reactive and tumor T_FH_ cells (A) Level of miR101 (has-miR-101) is low and similar in both tumor and reactive T_FH_ cells (p = 0,8 unpaired t-test, NS) (B).

Physiologically, in mice, Roquin limits ICOS expression by promoting the degradation of *ICOS* mRNA in a dose-dependent manner [24], [25]. In sanroque mice, mutated Roquin is unable to promote *ICOS* mRNA degradation, resulting in the overexpression of the protein. Here, we show that the level of *ICOS* mRNA expression is maintained even in the presence of *ROQUIN* transcripts both in human reactive and tumor T_FH_ cells (Figure 4A). This is in accordance with the common ICOS expression by neoplastic T-cells in AITL [26], [27]. It has been suggested that Roquin repressive effect on ICOS transcripts requires miR101 expression [25]. We therefore looked for miR101 expression in our T_FH_ cells. Level of miR101 was low and similar in both neoplastic and reactive T_FH_ cells (Figure 4B), in accordance with recent finding in mouse showing that BCL6 could repress inhibitors of specific T_FH_ expressing gene including miR101 [28].

## Conclusion

Altogether, by comparing reactive and AITL T_FH_ cells, we have shown here that neither alteration of *ROQUIN* gene nor deregulation of miR101 expression is likely to be a frequent recurrent abnormality in AITL. Expanding knowledge on the pathways deregulated by *Roquin* mutation in Sanroque mice might uncover other molecules of potential relevance to AITL pathophysiology.
